# Supplementary material for: Effect of antenatal depression on adverse birth outcomes in Gondar town, Ethiopia: A community-based cohort study
Source: PLoS One. 2020 Jun 17;15(6):e0234728. doi: 10.1371/journal.pone.0234728 (PMC7299401; doi:10.1371/journal.pone.0234728)
Supplement: Attachment — Submitted filename: Rebuttal letter.docx [file pone.0234728.s001.docx]

Date: 17/04/2020

**Rebuttal letter**

PONE-D-19-29080

Effect of Antenatal Depression on Adverse Birth Outcomes in Gondar Town, Ethiopia: A community-based cohort study

Abel Fekadu Dadi

**To PLoS one**

We appreciate the journal for its second quick review and decision process. We also would like to thank reviewers for their valuable comments. The revised manuscript would benefit from these comments and suggestions. We considered each comment and clarification questions of reviewers on the paper thoroughly. Our point-by-point responses for each feedback and questions are described in detail on the following pages. Further, the details of changes were shown by track changes in the supplementary document attached.

| **Reviewer #1:** | Author response |
| --- | --- |
| Abstract “The impact of psychosocial factors on pregnancy outcomes has been investigated in developed countries but few studies have been conducted in low-income countries.” This sentence does not look really important. If the authors have to report this, how can the outcome differ across culture? Why the HIC findings is not generalizable to LICs?  Introduction The authors argued that the moderating effect of depression was not studied in Ethiopia. If this is known in other countries, is it not generalizable to other countries like Ethiopia?    Methods The authors argued that preterm birth was not controlled in previous studies. it is understood that measurement of preterm birth is very problematic as women cannot remember the LMP which might have affected the results. The challenges related to assessment of preterm birth and low birth weight were not stated in limitations.  There is also stigma related to reporting stillbirth. How could the authors managed to reduce bias related to such stigma.  The grave limitation of previous studies on the association of depression and LBW were linked to low sample size which was also the case in the current study. How different is the current study from previous studies? | Thanks, reviewer.  Here we used this heading sentence to show that there is limited information in low-income countries about the association between antenatal depression and adverse birth outcomes. Now, we revised to show that antenatal depression significantly varies between high- and low-income countries and its effect would also alter accordingly, and this makes generalization difficult.  Thanks. Apart from the previously discussed difference in socio-economic and cultural differences between countries, there are also limited studies worldwide about the moderating and mediating effect of social support and stress coping ability of the association between antenatal depression and risk of adverse birth outcomes.  Thanks. Unfortunately, we did not say preterm birth was not controlled in previous studies, and the question is not clear. Perhaps, to minimize the measurement associated with LMP, we have also take ultrasonography information to decide. But this would not be true for all mothers, so the inclusion of the challenges related to measurement worth inclusion as a limitation. As such, we have included such problems associated with measurement in the limitation section accordingly.  In Ethiopia, the stigma associated with stillbirth is not known, or from my experience, there is no such stigma. As such issues are linked with religion, a community is far from believing that the problem is due to personal reasons unlike abortion, which is a serious socially unacceptable practice or event. Moreover, the data collectors were nurse health professionals, and they are the one routinely followed the mother’s pregnancy and birth. Therefore there is nothing private between the mothers and these nurse data collectors.  Thanks. Yes, such limitations are reported in previous studies. The case is also true in this study, specifically in moderation and mediation analysis where estimation was not possible because of small observations in a few categories of moderators. But the investigation on the direct association between antenatal depression and birth outcomes was not affected by sample size, unlike other previous studies as we can see from the width of confidence intervals. So, from our estimation, we could confidently report the lack of direct association between antenatal depression and adverse birth outcomes. Still, there is some sort of interaction observed between social support and stress coping ability, which its full investigation has been hampered due to small sample size in a few categories as summarized in the limitation section. |
| **Reviewer #2** |  |
| This paper was well conceived and well-written. The authors conducted a large survey of pregnant women (n~900) in Gondar town, Ethiopia. The main exposure in the study was antenatal depression and the main outcome was incidence of adverse birth outcome (preterm birth, low-birth weight or still birth). Information on a range of risk factors (e.g. occupation, age, marital status, religion, physical activity, social support, coping strategies, antenatal healthcare attendance etc.) was also collected and including in the data analysis (which was conducted using structural equation modelling). The authors report on the main risk factors for each adverse birth outcome.  The main findings in relation to the incidence of still birth indicates that Ethiopia is lagging behind other countries in reducing still births. Depression was not a risk factor for still birth in this study (perhaps due to low power); neither was is associated with low-birth weight or pre-term labour.  The main findings suggest that perceived partner support and ability to cope with stress were moderated the relationship between depression and low-birth weight (although the effect was small) - and the effect was stronger when considering preterm birth. There was no main effect for experiencing depression and low-birth weight or preterm birth. So the main take-home message appears to be that interventions efforts might be better focused on increasing partner support and the woman's ability to cope with stress.  The authors neatly report on the study strengths and limitations in the discussion. The survey response rate was extremely high(97.7%).  Comments/queries: Please provide more information on the collection of data during the second trimester. How regularly were women assessed between the second trimester and the outcome assessment (one month after birth). The data collection and the questionnaire section is somewhat confusing because it suggests that many of the study variables (e.g. partner support, stress coping ability) were assessed at the one-month post birth interview, as opposed to during the second trimester (i.e. during the pregnancy and before the birth outcome data was collected). Please clarify.  There is a bit of confusion in the discussion about the need for studies with larger sample sizes, although the large sample size of the study is listed as a study strength. It appears the authors mean that larger sample sizes are required to study rare outcomes (i.e. still birth) in future.  There appears to be a mix-up in the measurement section re the cutoff score for MUAC (18-22cm should be underweight and 22.5-31cm should be normal). | Thanks, so much reviewer for your encouragement. The paper is well understood.  Here indeed association found between antenatal depression and risk of stillbirth in the main model and not in the mediation analysis model. We hypothesized that this discrepancy would be because of a small number of stillbirths observed in this study.  Well understood.  Thanks so much.  Thanks, reviewer. All the covariates and the primary exposure variable (antenatal depression) were assessed during pregnancy. The outcome variables, stillbirth, preterm birth, and low birth weight were estimated within one month of delivery. Revised accordingly.  Thanks, reviewer. The sample size is relatively large. However, the exclusion of mothers with high risk (depression) because of ethical reasons would have affected the overall sample size (power) of the study. We also hypothesized that the marginally non-significant effect of antennal depression on stillbirth in mediation analysis is due to this exclusion. So, the possible solution for this rare outcome might be the use of case-control study or repeating the study by increasing sample size as this will increase the number of observed stillbirths.  Thanks, this has been revised. |
