# Supplementary material for: Effect of antenatal depression on adverse birth outcomes in Gondar town, Ethiopia: A community-based cohort study
Source: PLoS One. 2020 Jun 17;15(6):e0234728. doi: 10.1371/journal.pone.0234728 (PMC7299401; doi:10.1371/journal.pone.0234728)
Supplement: Attachment — Submitted filename: Rebuttal letter two.docx [file pone.0234728.s002.docx]

Date: 07/05/2020

**Rebuttal letter**

PONE-D-19-29080

Effect of Antenatal Depression on Adverse Birth Outcomes in Gondar Town, Ethiopia: A community-based cohort study

Abel Fekadu Dadi

**To PLoS one**

We appreciate the journal for its second quick review and decision process. On the second revision, one point raised, that is about the response rate.

**Question raised**: Please explain how did you achieve such high response rate on the survey, Thank you

**Answer**: though the response rate is claimed as high, according to the tight strategy we followed, for us, this is not actually high response rate. These were the strategies we followed to keep our response rate even more than higher than this.

1. Our data collectors were health extension workers (community health workers), who were female by gender and nurse by profession. They are assigned in the community to implement primary health care services at the household level. According to the Ethiopian government, one health extension worker has a mandate to cover at least 750 households in her catchment area. From the services, pregnancy follow up and management is their main role to reduce maternal mortality. So, in this study we used these community health workers, and the follow up and data collection was handy and easy for them. This majorly helped to maintain a high response rate and to reduce the lost to follow up in this study.
2. The intimacy and the trust that is available between health extension workers (data collectors) and pregnant mothers is also made the recruitment of women into the study and follow up very smooth. This also helps to maintain this high response rate.
3. The data collectors had list of pregnant mothers with their phone number and it was very easy to track and follow up by phone.
4. Due emphasis had been given during questionnaire development and questionnaires were made clear and short to minimize burden associated with the study participants.
5. Efforts had been made to make the objective, benefit, and risks associated with the study (actually no) clear to the study participants to have their commitment to maintain their involvement.
6. Strict supervision of the data collection process by supervisors and active involvement of the principal investigator in the supervision process could be also mentioned as a tool to maintain this response rate.
7. We used an online ecteronic based data collection technique which made the data collection very easy and handy for data collectors to minimize their burden, fatigue, and maintain their motivation throughout the data collection period.
